# Effect of maternal serum albumin level on birthweight and gestational age: an analysis of 39200 singleton newborns

**DOI:** 10.3389/fendo.2024.1266669

**Published:** 2024-03-05

**Authors:** Jiayi Wu, Xiaorui Liu, Chuanmei Qin, Jinwen Zhang, Xueqing Liu, Jianing Hu, Fan Wu, Cailian Chen, Yi Lin

**Affiliations:** ^1^ The International Peace Maternity and Child Health Hospital, School of Medicine, Shanghai Jiao Tong University, Shanghai, China; ^2^ Shanghai Key Laboratory of Embryo Original Diseases, School of Medicine, Shanghai Jiao Tong University, Shanghai, China; ^3^ Institute of Birth Defects and Rare Diseases, School of Medicine, Shanghai Jiao Tong University, Shanghai, China; ^4^ Department of Automation, Shanghai Jiao Tong University, Shanghai, China; ^5^ Key Laboratory of System Control and Information Processing, Ministry of Education of China, Shanghai, China; ^6^ Reproductive Medicine Center, Shanghai Jiao Tong University Affiliated Sixth People’s Hospital, Shanghai, China

**Keywords:** serum albumin, fetal growth, birthweight, gestational age, mid-term fetal growth

## Abstract

**Background:**

Serum albumin plays a pivotal role in regulating plasma oncotic pressure and modulating fluid distribution among various body compartments. Previous research examining the association between maternal serum albumin levels and fetal growth yielded limited and inconclusive findings. Therefore, the specific influence of serum albumin on fetal growth remains poorly understood and warrants further investigation.

**Methods:**

A retrospective study involved 39200 women who had a singleton live birth at a tertiary-care academic medical center during the period from January 2017 to December 2020. Women were categorized into four groups according to the quartile of albumin concentration during early pregnancy: Q1 group, ≤41.0 g/L; Q2 group, 41.1-42.6 g/L; Q3 group, 42.7-44.3 g/L and Q4 group, >44.3 g/L. The main outcome measures were mid-term estimated fetal weight, birthweight and gestational age. Multivariate linear and logistic regression analysis were performed to detect the independent effect of maternal serum albumin level on fetal growth after adjusting for important confounding variables.

**Results:**

In the crude analysis, a significant inverse correlation was found between early pregnancy maternal serum albumin levels and fetal growth status, including mid-term ultrasound measurements, mid-term estimated fetal weight, birthweight, and gestational age. After adjustment for a number of confounding factors, mid-term estimated fetal weight, birthweight, and birth height decreased significantly with increasing albumin levels. Compared to the Q2 group, the Q4 group had higher rates of preterm birth (aOR, 1.16; 95% CI, 1.01–1.34), small-for-gestational-age (aOR, 1.27; 95% CI, 1.11–1.45) and low birthweight (aOR, 1.41; 95% CI, 1.18–1.69), and lower rate of large-for-gestational-age (aOR, 0.85; 95% CI, 0.78–0.94). Moreover, to achieve the optimal neonatal outcome, women with higher early pregnancy albumin levels required a greater reduction in albumin levels in later pregnancy stages.

**Conclusions:**

A higher maternal serum albumin level during early pregnancy was associated with poor fetal growth, with the detrimental effects becoming apparent as early as the mid-gestation period. These findings provided vital information for clinicians to predict fetal growth status and identify cases with a high risk of adverse neonatal outcomes early on.

## Introduction

Optimal fetal growth and development are known to be the foundation for long-term human health, according to the well-known developmental origins of health and disease theory (1). Numerous studies have confirmed that intrauterine growth restriction or low birthweight can lead to diseases in children and adults, such as cognitive dysfunction and cardiovascular and metabolic diseases (2–4). More seriously, it is difficult to reverse the health status of an individual after birth, and some health defects may even cross generations (5). Although placental insufficiency, gestational hypertension, and preeclampsia have been reported to be risk factors for poor fetal growth (6, 7), its etiology is still unclear. A better understanding of modifiable factors associated with fetal growth would be vital to ensuring the maximum growth potential in early life.

Human serum albumin, an important indicator of nutritional status and hepatic function, is a widely used clinical marker. A lower serum albumin level may increase the risk of morbidity and mortality in both adults and children with various medical conditions, including stroke, renal disease, and malignancies (8). Notably, albumin, a major component of plasma proteins, plays a role in maintaining oncotic pressure and reflects plasma expansion (9). During pregnancy, inadequate plasma expansion is associated with the risk of low birthweight (LBW) (10), oligohydramnios (11), and preeclampsia (12). Growing evidence suggests that serum albumin levels could serve as an indicator of the risk and severity of preeclampsia (13, 14). Little is known, however, regarding the effect of maternal serum albumin on neonatal outcomes such as birthweight and birth length. Since albumin is a routine component of antenatal care, if proven to be an independent predictor for fetal growth, it could be a simple and low-cost method for early diagnosis of adverse neonatal outcomes.

To date, very few studies have examined the possible impact of maternal serum albumin on fetal growth (15–18), and their results are limited and contradictory. The main limitation to drawing robust and definitive conclusions is the absence of information on pregnancy complications, specifically gestational hypertension, preeclampsia, and gestational diabetes. Moreover, existing research were largely focused on women with term delivery, so the influence of maternal serum albumin levels on gestational age remains unknown. Therefore, there is clear need for a comprehensive investigation on the association between maternal serum albumin levels and fetal growth outcomes.

In the present study, we aimed to explore the impact of maternal serum albumin levels in early pregnancy on fetal growth by examining a large cohort of women with live-born singletons. Both neonatal outcomes and mid-term fetal growth were analyzed to predict the trajectory of fetal development throughout the duration of pregnancy, and the results offer crucial reference data for early interventions targeting inadequate fetal growth.

## Methods

### Study design and population

A retrospective study was conducted at the International Peace Maternity and Child Health Hospital (IPMCH) of Shanghai Jiao Tong University School of Medicine, a tertiary care hospital in China. The study protocol was approved by the Institute Medical Ethics Committee of IPMCH (reference number GKLW2021-17) and carried out according to the tenets of the Declaration of Helsinki. All women who had regular antenatal examination records and had a live birth (≥24 weeks of gestation) at IPMCH during January 2017 to December 2020 were included.

The exclusion criteria were: (1) multiple pregnancy, (2) *in vitro* fertilization, (3) maternal liver dysfunction (19), (4) maternal liver or renal disease, and (5) loss to follow-up or unavailability of main hepatic function records in the electronic database, including data on albumin, AST, and ALT levels.

### Data collection

The following demographic characteristics were extracted from the medical record system: maternal age, pre-pregnancy body mass index (BMI), gravidity, parity, education level, cigarette or alcohol consumption before pregnancy, medical history, pregnancy complications, ultrasound measurements, delivery method, gestational age, birthweight, birth length, and newborn sex. Gestational diabetes mellitus (GDM) was diagnosed based on a 2-h 75-g oral glucose tolerance test done at 24–28 weeks of gestation (20). Pregnancy-induced hypertension, including preeclampsia and gestational hypertension, was diagnosed based on diastolic blood pressure ≥90 mm Hg or systolic blood pressure ≥140 mm Hg measured twice after 20 weeks of gestation, with or without proteinuria. The records of liver biochemistry tests, including albumin, AST, and ALT levels, during the early pregnancy period (8–14 weeks of gestation) were measured by professional laboratory technicians, as previously described (19), and acquired from the hospital’s laboratory database. The reference normal range for alanine transaminase [ALT] and aspartate aminotransferase [AST] in the Chinese population is considered to be not exceeding 40 U/L for both enzymes (21). The normal local laboratory serum albumin level range is 35–52 g/L (17). Records of the maternal serum albumin level during the final antenatal examination prior to delivery were also obtained, and the change in albumin level was calculated as the albumin value from the last assessment minus the albumin value from early pregnancy.

Ultrasound measurements, including biparietal diameter (BPD), humerus length (HL), femur length (FL), head circumference (HC), and abdominal circumference (AC), were performed by highly trained and experienced sonographers using standard protocols and identical instruments. These biometric measurements were recorded during 21–23 weeks of gestation. BPD was defined as the linear distance from the outer edge of the proximal parietal bone to the inner edge of the distal parietal bone on a cross-section of the fetal brain. FL and HL were measured as the linear distance along the long axis of the femur and humerus, respectively. With the ellipse function of the ultrasonic equipment, HC was measured at the same level as BPD, while AC was measured in a plane perpendicular to the level of the fetal umbilical plexus. To better assess fetal growth during the second trimester, estimated fetal weight (EFW) was calculated using HC, AC, BPD, and FL, according to the Hadlock formula (22).

The primary outcomes were mid-term EFW, singleton birthweight, and gestational age. The secondary endpoints included birth length, birthweight z-score, rates of preterm birth (PTB), small-for-gestational-age (SGA), large-for-gestational-age (LGA), LBW, and macrosomia. Birthweight z-scores were computed based on a set of general population reference values for Chinese singleton births to correct for the effect of newborn gender and gestational age on birthweight (23). Macrosomia and LBW were defined as birth weight >4000 g and <2500 g, respectively. Very-small-for-gestational-age (VSGA), SGA, LGA, and very-large-for-gestational-age (VLGA) were defined as birthweight <3^rd^, <10^th^, >90^th^, and >97^th^ percentiles for gestational age, respectively. Very preterm birth (VPTB) and PTB were defined as delivery at <32 gestational weeks and <37 gestational weeks, respectively.

### Statistical analysis

Continuous variables were described as mean values with standard deviations and compared by one-way analysis of variance. Categorical variables were presented as numbers with corresponding percentages and compared by Fisher’s exact test or Pearson’s chi-squared test, as appropriate. Following a methodology similar to previous literature (17), the women were further divided into four groups based on their albumin concentration: Q1, ≤41.0 g/L; Q2, 41.1–42.6 g/L; Q3, 42.7–44.3 g/L; and Q4, >44.3 g/L. The Q2 group was used as the reference for all comparisons. This grouping approach was implemented to facilitate a more comprehensive and detailed analysis of the influence of varying albumin levels on birth outcomes. Multivariable linear and logistic regression were performed to explore the association of maternal serum albumin level with fetal growth status after adjusting for several potential confounders, including maternal age, BMI, gravidity, parity, educational level, alcohol and cigarette consumption before pregnancy, ALT, AST, gestational age at sampling, pregnancy-induced hypertension, and GDM. The inclusion of potential confounders was determined based on the results of univariate and stepwise regression combined with variables related to serum albumin and neonatal outcomes indicated in previous studies. To further explore the dose-response association between serum albumin level and fetal growth, spline smoothing (24) on the basis of a generalized additive model were performed after adjustment for confounding factors. Given that serum albumin levels may vary as pregnancy progresses, to further minimize biases that may be caused by measurement of albumin levels at different gestational weeks, a sensitivity analysis was performed using women with a sampling time of 12 gestational weeks. Within this study cohort, the number of measurements conducted at 12 weeks was the highest, comprising 49% of the total population. All statistical analyses were performed using SAS software, version 9.4 (SAS Institute). Two-tailed P values <0.05 were considered to indicate significance.

## Results

A total of 62299 women with a singleton live birth were selected from our electronic database, of which 39200 women fulfilling the inclusion criteria of the study were finally included. The details of the participant selection process are displayed in **Supplementary Figure 1**. A comparative analysis of the demographic characteristics was performed between the excluded and included participants (**Supplementary Table 1**).

The baseline demographic and clinical characteristics of the study population are presented in **Table 1**. In brief, the mean maternal age and BMI were 31.11 ± 3.88 years and 21.24 ± 2.82 kg/m^2^, respectively. Of all the women, 26902 (68.6%) were primipara, and 22235 (56.7%) underwent vaginal delivery. The mean albumin level during early pregnancy was 42.68 ± 2.51 g/L, with the mean ALT level and AST level being 16.03 ± 14.10 U/L and 18.43 ± 7.62 U/L, respectively. Hepatic function measurements were taken at 11.96 ± 1.10 gestational weeks. The proportions of women diagnosed with pregnancy-induced hypertension and GDM were 4.8% and 14.3%, respectively.

**Table 1:** Basic characteristics of study population.

Characteristics	Participants, No. (%)
N	39200
Age (years)	31.11 ± 3.88
BMI (kg/m^2^)	21.24 ± 2.82
Gravidity	
0	19037 (48.6)
≥1	20163 (51.4)
Parity	
0	26902 (68.6)
≥1	12298 (31.4)
Educational level	
Below college degree	10183 (26.0)
Bachelor’s degree	20402 (52.0)
Master’s or PHD degree	8615 (22.0)
Drinking before pregnancy (yes)	581 (1.5)
Smoking before pregnancy (yes)	228 (0.6)
Albumin (g/L)	42.68 ± 2.51
Alanine transaminase (U/L)	16.03 ± 14.10
Aspartate aminotransferase (U/L)	18.43 ± 7.62
Gestational age at sampling (weeks)	11.96 ± 1.10
Change in albumin level (g/L)	-6.67 ± 2.80
Delivery method	
Vaginal	22235 (56.7)
Cesarean	16965 (43.3)
Hypertension	
Pregnancy induced	1845 (4.8)
Preexisting	695 (1.8)
Diabetes	
Pregnancy induced	5620 (14.3)
Preexisting	94 (0.2)

Data are presented as mean ± SD for continuous variables and n (%) for dichotomous variables.

With regard to the stratification of women into groups according to the quartiles of albumin concentration, 10269, 9741, 9707, and 9483 women were assigned to Q1, Q2, Q3, and Q4 groups, respectively. The baseline characteristics across the Q1-Q4 groups were shown in **Supplementary Table 2**. The associations between maternal serum albumin level and fetal growth are shown in **Table 2**. Women with higher levels of albumin had significantly lower BPD, HL, FL, HC, AC, and EFW in the second trimester (P < 0.001 for all). With regard to the neonatal outcomes, participants in the Q4 group (the highest quartile of albumin concentration) had significantly lower birthweight, birthweight z-scores, and birth length (P < 0.001). With increase in albumin concentrations, that is, from Q1 to Q4, the rates of PTB, LBW, SGA, and VSGA significantly increased (P = 0.036 for PTB; P < 0.001 for the rest), whereas the rates of macrosomia, LGA, and VLGA decreased (P < 0.001 for all). Stratified analyses of birthweight and birth length were further conducted based on the newborns’ gender. The results demonstrated that, regardless of gender, birthweight, birthweight z-score, and birth length decreased with increasing serum albumin levels (**Supplementary Table 3**).

**Table 2:** Main fetal growth parameters of live born singletons stratified by quartiles of maternal albumin.

	Total (n=39200)	Quartiles of albumin level				P value
		Q1 (n=10269)	Q2 (n=9741)	Q3 (n=9707)	Q4 (n=9483)	
Mid-term fetal growth						
Biparietal diameter (mm)	55.81 ± 3.01	55.92 ± 3.00	55.91 ± 3.03	55.79 ± 3.02	55.60 ± 2.96	<0.001
Humerus length (mm)	36.22 ± 2.08	36.37 ± 2.08	36.33 ± 2.09	36.16 ± 2.06	36.01 ± 2.05	<0.001
Femur length (mm)	38.22 ± 2.19	38.38 ± 2.20	38.33 ± 2.21	38.15 ± 2.18	37.99 ± 2.13	<0.001
Head circumference (mm)	198.43 ± 9.57	198.66 ± 9.50	198.68 ± 9.71	198.48 ± 9.48	197.88 ± 9.57	<0.001
Abdominal circumference (mm)	173.81 ± 10.55	174.77 ± 10.80	174.28 ± 10.50	173.61 ± 10.51	172.50 ± 10.21	<0.001
Estimated fetal weight (g)	494.87 ± 63.78	500.75 ± 65.77	498.2 ± 64.29	493.28 ± 62.96	486.72 ± 60.93	<0.001
Neonatal outcomes						
Gestational age (weeks)	38.96 ± 1.36	38.92 ± 1.33	38.97 ± 1.33	39.00 ± 1.37	38.96 ± 1.38	0.001
PTB	1771 (4.5)	432 (4.2)	413 (4.2)	476 (4.9)	450 (4.7)	0.036
VPTB	124 (0.3)	35 (0.3)	30 (0.3)	28 (0.3)	31 (0.3)	0.921
Male sex	20014 (51.1)	4978 (48.5)	5067 (52.0)	4990 (51.4)	4979 (52.5)	<0.001
Body length (cm)	49.82 ± 1.36	49.89 ± 1.30	49.83 ± 1.34	49.82 ± 1.37	49.76 ± 1.41	<0.001
Birthweight (g)	3333.91 ± 430.08	3366.77 ± 425.99	3345.25 ± 426.32	3322.94 ± 430.74	3297.88 ± 434.51	<0.001
Birthweight z-score	0.22 ± 0.94	0.31 ± 0.95	0.24 ± 0.94	0.18 ± 0.94	0.12 ± 0.94	<0.001
LBW	1024 (2.6)	211 (2.1)	237 (2.4)	260 (2.7)	316 (3.3)	<0.001
Macrosomia	2097 (5.3)	620 (6.0)	530 (5.4)	505 (5.2)	442 (4.7)	<0.001
SGA	1899 (4.8)	407 (4.0)	438 (4.5)	485 (5.0)	569 (6.0)	<0.001
LGA	4863 (12.4)	1501 (14.6)	1229 (12.6)	1133 (11.7)	1000 (10.5)	<0.001
VSGA	458 (1.2)	92 (0.9)	98 (1.0)	118 (1.2)	150 (1.6)	<0.001
VLGA	1506 (3.8)	486 (4.7)	376 (3.9)	349 (3.6)	295 (3.1)	<0.001

All ultrasound measurements were performed at 21-23 weeks of gestation. Estimated fetal weight was computed from biparietal diameter, femur length, head circumference and abdominal circumference using a Hadlock formula. Data are presented as mean ± SD for continuous variables and n (%) for dichotomous variables.

Multiple linear regression analyses were run to explore the relationship between albumin level and fetal growth (**Table 3**). According to the results of the fully adjusted analysis, compared with participants in the second quartile, those in the higher quartiles of albumin level had significantly lower EFW during the second trimester (Q3: β = -4.18, 95% CI = -5.97 to -2.39, P < 0.001; Q4: β = -9.36, 95% CI, -11.23 to -7.49; P < 0.001). Infants with higher maternal albumin levels had significantly lower birth weights than those in the second quartile, with the following values of adjusted regression coefficients: Q3: β = -17.32, 95% CI = -29.23 to -5.41 (P = 0.004); Q4: β = -36.89, 95% CI = -49.32 to -24.45 (P < 0.001). In addition, elevated albumin levels were associated with a decrease in gestational age-adjusted birthweight z-score and birth length after adjustment for confounding factors.

**Table 3:** Results of multiple linear regression analysis of main fetal growth parameters among live born singletons.

	Q1		Q2	Q3		Q4	
	β (95% CI)	P value		β (95% CI)	P value	β (95% CI)	P value
Mid-term ultrasound measurements							
Biparietal diameter	0.00(-0.01,0.01)	0.364	Reference	-0.01(-0.02,-0.00)	0.004	-0.03(-0.04,-0.02)	<0.001
Humerus length	0.00(-0.00,0.01)	0.803	Reference	-0.02(-0.02,-0.01)	<0.001	-0.03(-0.03,-0.02)	<0.001
Femur length	0.00(-0.00,0.01)	0.335	Reference	-0.02(-0.02,-0.01)	<0.001	-0.03(-0.04,-0.02)	<0.001
Head circumference	-0.00(-0.03,0.02)	0.813	Reference	-0.02(-0.05,0.01)	0.140	-0.08(-0.11,-0.05)	<0.001
Abdominal circumference	0.03(-0.00,0.06)	0.079	Reference	-0.05(-0.08,-0.02)	0.001	-0.14(-0.17,-0.11)	<0.001
Estimated fetal weight	1.41(-0.37,3.20)	0.119	Reference	-4.18(-5.97,-2.39)	<0.001	-9.36(-11.23,-7.49)	<0.001
Neonatal outcomes							
Gestational age	0.01(-0.03,0.05)	0.592	Reference	-0.00(-0.04,0.03)	0.909	-0.03(-0.07,0.00)	0.082
Body length	0.04(0.01,0.08)	0.024	Reference	-0.01(-0.05,0.03)	0.630	-0.06(-0.10,-0.02)	0.006
Birthweight	9.12(-2.72,20.96)	0.131	Reference	-17.32(-29.23,-5.41)	0.004	-36.89(-49.32,-24.45)	<0.001
Birthweight z-score	0.03(0.00,-0.06)	0.023	Reference	-0.04(-0.06,-0.01)	0.004	-0.09(-0.11,-0.06)	<0.001

Analyses were adjusted for age, BMI, gravidity, parity, educational level, alcohol and cigarette consumption before pregnancy, ALT, AST, gestational age at sampling, pregnancy induced hypertension and gestational diabetes mellitus.

As shown in **Table 4**, after adjustment for several confounding variables, the highest quartile of albumin values was associated with higher risks of PTB (adjusted odds ratio (aOR) = 1.16; 95% CI = 1.01–1.34; P = 0.038), LBW (aOR = 1.41; 95% CI = 1.18–1.69; P < 0.001) and SGA (aOR = 1.27; 95% CI = 1.11–1.45; P = 0.001) than the second quartile. Furthermore, the fourth quartiles were associated with lower rates of LGA (aOR = 0.85, 95% CI = 0.78–0.94, P = 0.001) than the second quartile. Given the gradual decline of maternal albumin levels during pregnancy, we strategically selected women with a sampling time of 12 weeks for sensitivity analysis to minimize potential sampling time bias. A total of 19189 women were included in the sensitivity analysis, and the findings remained consistent with previous observations, affirming the stability and reliability of the results (**Supplementary Table 4**).

**Table 4:** Crude and adjusted ORs for adverse neonatal outcomes in singleton births by maternal albumin levels.

	Q1	P value	Q2	Q3	P value	Q4	P value
PTB							
OR (95% CI)	0.99 (0.86,1.14)	0.903	Reference	1.17 (1.02,1.33)	0.027	1.13 (0.98,1.29)	0.092
AOR (95% CI)	0.95 (0.83,1.10)	0.502	Reference	1.21 (1.05,1.38)	0.007	1.16 (1.01,1.34)	0.038
LBW							
OR (95% CI)	0.84 (0.70,1.01)	0.070	Reference	1.10 (0.92,1.32)	0.279	1.38 (1.17,1.64)	<0.001
AOR (95% CI)	0.85 (0.71,1.04)	0.108	Reference	1.14 (0.95,1.36)	0.173	1.41 (1.18,1.69)	<0.001
Macrosomia							
OR (95% CI)	1.12 (0.99,1.26)	0.062	Reference	0.96 (0.85,1.09)	0.497	0.85 (0.75,0.97)	0.016
AOR (95% CI)	1.03 (0.91,1.17)	0.604	Reference	0.97 (0.86,1.11)	0.670	0.88 (0.77,1.01)	0.072
SGA							
OR (95% CI)	0.88 (0.76,1.01)	0.060	Reference	1.12 (0.98,1.28)	0.102	1.36 (1.19,1.54)	<0.001
AOR (95% CI)	0.98 (0.85,1.13)	0.755	Reference	1.08 (0.95,1.24)	0.246	1.27 (1.11,1.45)	0.001
LGA							
OR (95% CI)	1.19 (1.10,1.29)	<0.001	Reference	0.92 (0.84,1.00)	0.048	0.82 (0.75,0.89)	<0.001
AOR (95% CI)	1.09 (1.00,1.18)	0.051	Reference	0.94 (0.86,1.02)	0.149	0.85 (0.78,0.94)	0.001

Analyses were adjusted for age, BMI, gravidity, parity, educational level, alcohol and cigarette consumption before pregnancy, ALT, AST, gestational age at sampling, pregnancy induced hypertension and gestational diabetes mellitus. OR Odd ratio, AOR adjusted odd ratio.

**Figure 1:**
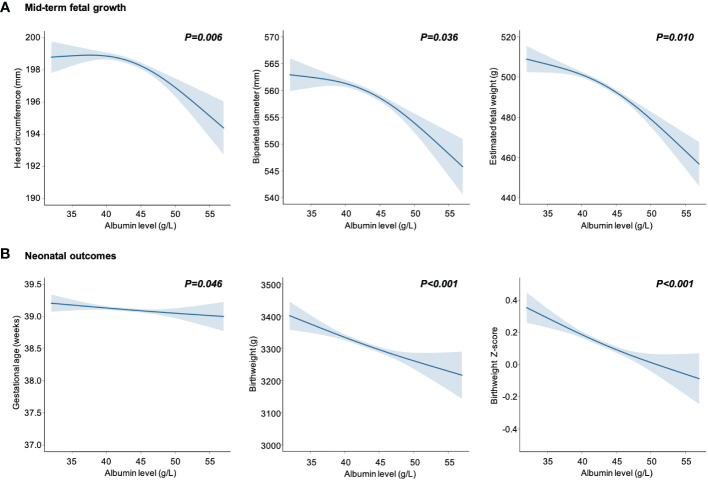
Maternal serum albumin in relation to mid-term fetal growth **(A)** and neonatal outcomes **(B)**. Data are presented as estimated mean with 95% CIs (shaded areas), adjusted for age, body mass index, gravidity, parity, educational level, alcohol and cigarette consumption before pregnancy, alanine transaminase, aspartate aminotransferase, gestational age at sampling, pregnancy induced hypertension and gestational diabetes mellitus.

The dose–response relationships between maternal albumin levels and fetal growth are displayed visually in **Figure 1**. After adjusting for various confounding factors, there was a notable decline observed in mid-term HC, BPD, and EFW measurements in women with elevated maternal albumin levels (**Figure 1A**). Furthermore, a significant inverse correlation was found between maternal serum albumin levels and neonatal growth status, including birthweight and birthweight z-score (**Figure 1B**). Gestational age showed a decrease in correlation with increasing levels of maternal albumin, albeit of a minor magnitude.

**Figure 2:**
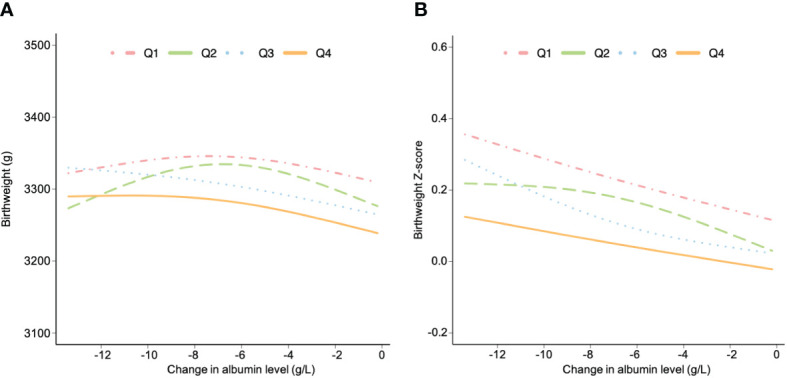
The associations between change in albumin level and birthweight **(A)** and birthweight z-score **(B)** stratified by albumin level in early pregnancy. Data are presented as estimated mean, adjusted for age, body mass index, gravidity, parity, educational level, alcohol and cigarette consumption before pregnancy, alanine transaminase, aspartate aminotransferase, gestational age at sampling and pregnancy induced hypertension and gestational diabetes mellitus. All women were grouped based on the quartile of albumin level during early pregnancy (Q1, Q2, Q3 and Q4).

The associations of change in albumin level with birthweight and birthweight z-score were analyzed. The population were categorized into four groups based on the quartiles of early pregnancy albumin concentration (Q1-Q4). The observation reveals that the Q4 group required a more significant decline in albumin levels in the later pregnancy stages to achieve the same birthweight and z-score as the Q1 group. (**Figure 2**). Additionally, women in the Q4 group showed lower potential for fetal development in terms of birthweight compared to women in the Q1 group. (**Figure 2**).

## Discussion

In this large cohort study, we investigated the impact of maternal serum albumin level on fetal growth in 39200 women with live-born singletons. Our findings indicated that a high maternal serum albumin level during early pregnancy was associated with poor fetal growth, with the detrimental effects becoming apparent as early as mid-gestation. Moreover, women with elevated albumin levels in early pregnancy required a more significant reduction in albumin levels during later stages of gestation to attain the optimal birth outcome. These findings provide vital information that could help clinicians to predict fetal growth velocity and identify cases with a high risk of adverse neonatal outcomes early in pregnancy.

To date, only a few studies have focused on the potential association between maternal albumin levels and fetal growth, and two earlier small-scale studies failed to find any correlation between neonatal outcomes and albumin concentrations (15, 18). In 1996, Hasin and colleagues analyzed 151 pregnant women from poor urban communities and found that serum albumin levels were significantly lower in the women who had low birthweight infants than in those who had normal weight infants (16). This was subsequently challenged by a prospective study, which reported an inverse association between maternal serum albumin level and birthweight when the measurements were done during the third trimester (25). However, during early pregnancy, no significant correlation was observed between maternal albumin levels and birthweight (25). More recently, a study involving 3065 term-born singletons revealed a reverse U-shaped relationship between the mid-trimester albumin level and fetal growth (17). Nevertheless, as the authors acknowledged, the missing data on pregnancy complications, such as GDM, limited the accuracy of their results (16).

Of note, gestation-adjusted z-scores were not calculated in the aforementioned literatures. Since the mean birthweight varied with race and region (26, 27), it is difficult to compare these studies. Moreover, none of these studies considered the possible adverse effects of pregnancy-induced hypertension, preeclampsia, and GDM on fetal growth. Thus, the reliability of the studies reported so far on this topic is limited. Most importantly, while anthropometric measurements taken at birth can reflect the culminative effect of an aberrant intrauterine environment on fetal growth, it does not provide a picture of specific changes in the fetal growth trajectory.

The present study, aiming to improve on the limitations of the previous studies described above, examined the precise role of maternal serum albumin level in fetal growth. Our results, based on the records of 39200 women with live-born singletons, clearly demonstrated that a high maternal serum albumin level had an adverse impact on fetal growth that initiated during the mid-gestational period and enduring until the late stages of pregnancy.

The reason why a high maternal serum albumin level leads to poor fetal growth is unclear. It is speculated that the resulting difference in plasma volume may play a role. Plasma volume expansion is a central physiological regulatory mechanism in pregnancy that begins as early as 6 to 8 gestational weeks. Although the mechanism underlying its role remains unclear, it has been suggested that reduced blood viscosity may favor blood flow in the maternal intervillous space (28). In addition, hemodilution in pregnancy is believed to prevent thrombosis in the uteroplacental circulation and further promote fetal development (29). Thus, plasma volume expansion is important for fetal growth. In fact, there is growing evidence that inadequate plasma volume expansion is associated with increased rates of intrauterine growth restriction and PTB (12, 30). This implies that the failure of plasma volume expansion in women with high levels of serum albumin may have implications for fetal growth and ultimately influence birthweight of infants. In the context of laboratory preparation for biochemical assays, the introduction of an anticoagulant, followed by centrifugation, yields a specimen known as plasma. Conversely, when an anticoagulant is omitted, blood naturally coagulates, resulting in the formation of serum upon centrifugation. Plasma typically exhibits a total protein concentration that is approximately 0.2-0.5g/dL higher than that of serum. This distinction primarily arises from fibrinogen, a protein component that is consumed during the coagulation process. Despite the minor difference in total protein concentration between plasma and serum, clinically significant serum albumin levels provide a direct reflection of changes in plasma albumin concentration. Consequently, serum albumin levels serve as an indicator of how pregnant women respond to fluctuations in plasma volume throughout various stages of pregnancy.

Previous studies on this topic were largely carried out in women with term delivery, thus the possible influence of maternal albumin level on gestational age could not be fully explored. Only one study demonstrated a weak positive correlation between mid-pregnancy albumin level and birth duration, but it was limited by the absence of data on confounding factors (25). Contrary to their results, the current study found that elevated albumin levels were associated with decreased gestational age after adjusting for potential confounders. Also, women in the third and fourth quartiles were associated with a higher PTB rate compared to the lowest quartile.

Detailed information on fetal development across pregnancy trimesters provides important information for clinical practice. Ultrasound measurements during mid-pregnancy can reflect fetal growth and the internal processes during the entire duration of pregnancy. Hence, it elucidates the underlying biological mechanisms that contribute to the observed association between maternal serum albumin levels and fetal growth. Therefore, it is of vital importance to further explore the fetal growth trajectory with the use of ultrasound measures during pregnancy. The results of the current study indicated that albumin levels were inversely related to BPD, HL, FL, HC, and AC, which may have an influence on the subsequent health and development of the infant. For instance, BPD and HC, as indicators of head size, are correlated with cognitive achievement during childhood (31). Further, AC is a critical indicator of fetal liver size and subcutaneous fat deposition, and is also associated with cardiometabolic status later on in life (32), and FL has been reported to be associated with economic productivity in adulthood (33). In the present study, impaired fetal development appeared as early as the second trimester and persisted until delivery. Thus, to prevent poor fetal growth, it is of vital importance to monitor serum albumin levels from early pregnancy and carry out appropriate clinical interventions simultaneously. Such monitoring can serve as an invaluable tool for both doctors and pregnant women in identifying pregnancy-related risks at an early stage, facilitating timely interventions for enhanced outcomes. For the treatment of hypoproteinemia, if there are no contraindications related to the primary disease, a high-protein, high-calorie diet can be administered, ensuring an appropriate protein intake, providing sufficient calorie supply, and simultaneously supplementing with an adequate amount of vitamins.

During pregnancy, there is a normal reduction of serum albumin concentrations along with the increase of plasma volume (25). Our study, for the first time, discovered an inverted U-shaped relationship between change in albumin level and fetal development. The results demonstrated that both excessive and inadequate reductions in albumin levels had adverse effects on fetal development. For women with higher albumin levels in early pregnancy, it was necessary for them to further reduce their albumin levels during subsequent pregnancy stages in order to reach the highest developmental potential. The data from this study highlighted the importance of monitoring maternal serum albumin levels throughout the entire duration of pregnancy.

The main strength of the current study was the large sample size. To the best of our knowledge, this is the largest study assessing the effect of maternal serum albumin level on fetal growth. Another strength is that a number of relevant confounders that might otherwise have caused a bias in the results were adjusted for in this study, especially pregnancy complications. Moreover, ultrasound measurements during pregnancy combined with birth weight assessments were used to evaluate the trajectory of fetal growth, and this provided a better understanding of the underlying mechanisms that potentially drive the observed associations. Additionally, the laboratory conditions and clinical procedures remained unchanged during the study period. For example, the ultrasound measurements were performed by the same group of trained sonographers, and this reduced any intra-observer variability. Despite these advantages, the present study is limited by its retrospective nature. In our study, we excluded cases with missing essential data, such as albumin levels, birthweight and gestational age, which may introduce some degree of bias. To address this concern, we conducted a comparative analysis of baseline characteristics between the excluded and included cases. The results revealed that the excluded group had, on average, a slightly higher age (0.5 years) and a slightly higher BMI (0.1 kg/m2) compared to the included group. Furthermore, the excluded group exhibited a higher proportion of cesarean section deliveries and a greater incidence of gestational diabetes and pregnancy-induced hypertension. This may be attributed to a significant portion of the excluded population undergoing assisted reproductive technology (9839 individuals), a group characterized by older age and a higher incidence of pregnancy complications (34, 35). In addition, in an attempt to overcome any biases associated with the study design, we carefully reviewed the data according to strict criteria and conducted sensitivity analysis to reinforce the robustness of our findings.

## Conclusions

In conclusion, the present large retrospective study showed that a high maternal serum albumin concentration was associated with impaired fetal growth in singletons. Thus, maternal serum albumin level may be used as an indicator in the clinic, based on which maximum fetal growth potential can be maintained in early pregnancy. Further studies are needed to verify our results.

## Data availability statement

The raw data supporting the conclusions of this article will be made available by the authors, without undue reservation.

## Ethics statement

The studies involving humans were approved by Institute Medical Ethics Committee of the International Peace Maternity and Child Health Hospital. The studies were conducted in accordance with the local legislation and institutional requirements. The participants provided their written informed consent to participate in this study.

## Author contributions

JW: Writing – original draft. XiL: Writing – review & editing. CQ: Formal analysis, Writing – original draft. JZ: Formal analysis, Writing – original draft. XuL: Formal analysis, Writing – original draft. JH: Formal analysis, Writing – original draft. FW: Formal analysis, Writing – review & editing. CC: Project administration, Writing – review & editing. YL: Conceptualization, Funding acquisition, Writing – original draft.
