# Supplementary material for: Effect of maternal serum albumin level on birthweight and gestational age: an analysis of 39200 singleton newborns
Source: Front Endocrinol (Lausanne). 2024 Mar 5;15:1266669. doi: 10.3389/fendo.2024.1266669 (PMC10948486; doi:10.3389/fendo.2024.1266669)
Supplement: Supplementary file 1 [file Image_1.pdf]

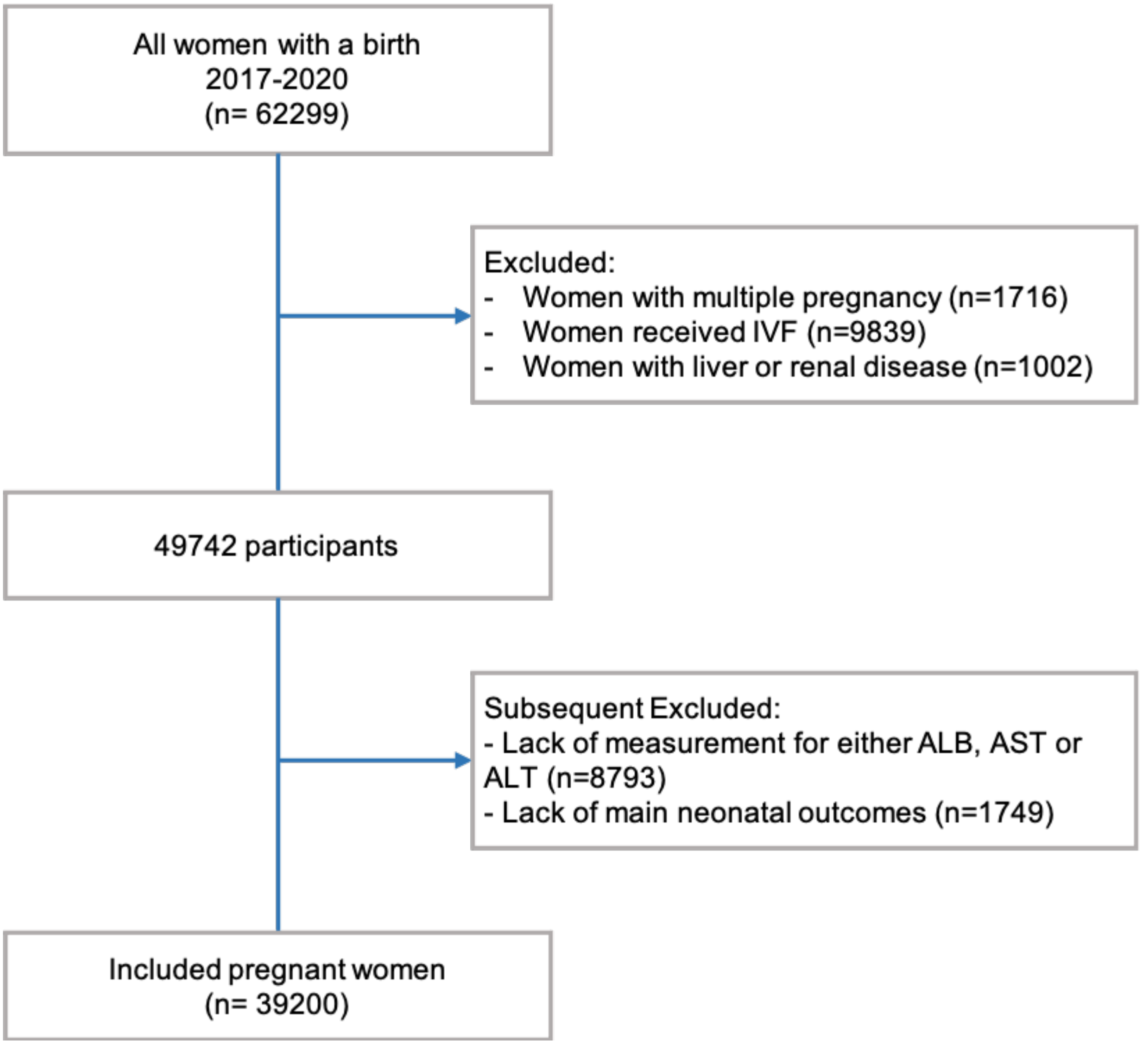

Supplementary Figure 1 Flow diagram for the selection of subjects for the study from the cohort. IVF, in vitro fertilization; ALT, alanine transaminase; AST, aspartate aminotransferase.
