# Supplementary material for: Effect of maternal serum albumin level on birthweight and gestational age: an analysis of 39200 singleton newborns
Source: Front Endocrinol (Lausanne). 2024 Mar 5;15:1266669. doi: 10.3389/fendo.2024.1266669 (PMC10948486; doi:10.3389/fendo.2024.1266669)
Supplement: Supplementary file 2 [file Table_1.docx]

**Supplementary Table 1 Basic characteristics of included participants and excluded participants.**

| Characteristics | Included Participants | Excluded Participants | P value |
| --- | --- | --- | --- |
| N | 39200 | 23099 |  |
| Age (years) | 31.11±3.88 | 31.64±4.23 | <0.001 |
| BMI (kg/m^2^) | 21.24±2.82 | 21.34±2.91 | <0.001 |
| Gravidity |  |  |  |
| 0 | 19037(48.6) | 11098(48.0) | 0.193 |
| ≥1 | 20163(51.4) | 12001(52.0) |  |
| Parity |  |  |  |
| 0 | 26902(68.6) | 15615(67.6) | 0.008 |
| ≥1 | 12298(31.4) | 8091(32.4) |  |
| Educational level |  |  |  |
| Below college degree | 10183(26.0) | 10531(45.6) | <0.001 |
| Bachelor’s degree | 20402(52.0) | 9052 (39.2) |  |
| Master’s or PHD degree | 8615(22.0) | 3516(15.2) |  |
| Drinking before pregnancy (yes) | 581(1.5) | 346(1.5) | 0.875 |
| Smoking before pregnancy (yes) | 228(0.6) | 106(0.5) | 0.043 |
| Delivery method |  |  |  |
| Vaginal | 22235(56.7) | 11472(49.7) | <0.001 |
| Cesarean | 16965(43.3) | 11627(50.3) |  |
| Hypertension |  |  |  |
| Pregnancy induced | 1845(4.8) | 1591(6.9) | <0.001 |
| Preexisting | 695(1.8) | 464(2.0) | 0.035 |
| Diabetes |  |  |  |
| Pregnancy induced | 5620(14.3) | 4316(18.7) | <0.001 |
| Preexisting | 94(0.2) | 45(0.2) | 0.250 |

Data are presented as mean ± SD for continuous variables and n (%) for dichotomous variables.
