# Supplementary material for: Effect of maternal serum albumin level on birthweight and gestational age: an analysis of 39200 singleton newborns
Source: Front Endocrinol (Lausanne). 2024 Mar 5;15:1266669. doi: 10.3389/fendo.2024.1266669 (PMC10948486; doi:10.3389/fendo.2024.1266669)
Supplement: Supplementary file 3 [file Table_2.docx]

**Supplementary Table 2 Basic characteristics of study population stratified by quartiles of maternal albumin.**

| Characteristics | Q1 | Q2 | Q3 | Q4 | P value |
| --- | --- | --- | --- | --- | --- |
| N | 10269 | 9741 | 9707 | 9783 |  |
| Age (years) | 32.30±4.09 | 31.18±3.86 | 30.65±3.69 | 30.21±3.51 | <0.001 |
| BMI (kg/m^2^) | 21.53±2.93 | 21.25±2.76 | 21.16±2.80 | 21.01±2.76 | <0.001 |
| Gravidity |  |  |  |  |  |
| 0 | 4526(44.1) | 4599(47.2) | 5228(53.9) | 4684(49.4) | <0.001 |
| ≥1 | 5743(55.9) | 5142(52.8) | 4479(46.1) | 4799(50.6) |  |
| Parity |  |  |  |  |  |
| 0 | 6205(60.4) | 6573(67.5) | 7295(75.2) | 6829(72.0) | <0.001 |
| ≥1 | 4064(39.6) | 3168(32.5) | 2412(24.8) | 2654(28.0) |  |
| Educational level |  |  |  |  |  |
| Below college degree | 2812(27.4) | 2528(26) | 2457(25.3) | 2386(25.2) | <0.001 |
| Bachelor’s degree | 5163(50.3) | 5133(52.7) | 5071(52.2) | 5035(53.1) |  |
| Master’s or PHD degree | 2294(22.3) | 2080(21.4) | 2179(22.4) | 2062(21.7) |  |
| Drinking before pregnancy (yes) | 158(1.5) | 163(1.7) | 127(1.3) | 133(1.4) | 0.166 |
| Smoking before pregnancy (yes) | 67(0.7) | 61(0.6) | 54(0.6) | 46(0.5) | 0.413 |
| Delivery method |  |  |  |  |  |
| Vaginal | 5494(53.5) | 5469(56.1) | 5695(58.7) | 5577(58.8) | <0.001 |
| Cesarean | 4775(46.5) | 4272(43.9) | 4012(41.3) | 3906(41.2) |  |
| Hypertension |  |  |  |  |  |
| Pregnancy induced | 402(3.9) | 469(4.8) | 465(4.8) | 509(5.4) | <0.001 |
| Preexisting | 135(1.3) | 139(1.4) | 165(1.7) | 256(2.7) | <0.001 |
| Diabetes |  |  |  |  |  |
| Pregnancy induced | 1468(14.3) | 1394(14.3) | 1444(14.9) | 1314(13.9) | 0.251 |
| Preexisting | 28(0.3) | 22(0.2) | 16(0.2) | 28(0.3) | 0.292 |

Data are presented as mean ± SD for continuous variables and n (%) for dichotomous variables.
