# Supplementary material for: Effect of maternal serum albumin level on birthweight and gestational age: an analysis of 39200 singleton newborns
Source: Front Endocrinol (Lausanne). 2024 Mar 5;15:1266669. doi: 10.3389/fendo.2024.1266669 (PMC10948486; doi:10.3389/fendo.2024.1266669)
Supplement: Supplementary file 4 [file Table_3.docx]

**Supplementary Table 3 Main fetal growth parameters of live born singletons** **stratified by quartiles of maternal albumin and genders.**

|  | **Q1** | **Q2** | **Q3** | **Q4** | **P value** |
| --- | --- | --- | --- | --- | --- |
| **Male** | N=4978 | N=5067 | N=4990 | N=4979 |  |
| Body length (cm) | 50.02±1.31 | 49.95±1.42 | 49.95±1.38 | 49.9±1.38 | <0.001 |
| Birthweight (g) | 3419.89±432 | 3394.22±440.24 | 3370.82±435.1 | 3347.52±437.14 | <0.001 |
| Birthweight z-score | 0.34±0.93 | 0.26±0.94 | 0.19±0.93 | 0.14±0.94 | <0.001 |
| **Female** | N=5291 | N=4674 | N=4717 | N=4504 |  |
| Body length (cm) | 49.76±1.28 | 49.7±1.23 | 49.67±1.34 | 49.61±1.44 | <0.001 |
| Birthweight (g) | 3316.8±414.13 | 3292.17±404.09 | 3272.3±420.24 | 3243.01±424.94 | <0.001 |
| Birthweight z-score | 0.29±0.96 | 0.21±0.93 | 0.17±0.94 | 0.1±0.94 | <0.001 |

Data are presented as mean ± SD for continuous variables.
