# Supplementary material for: Effect of maternal serum albumin level on birthweight and gestational age: an analysis of 39200 singleton newborns
Source: Front Endocrinol (Lausanne). 2024 Mar 5;15:1266669. doi: 10.3389/fendo.2024.1266669 (PMC10948486; doi:10.3389/fendo.2024.1266669)
Supplement: Supplementary file 5 [file Table_4.doc]

**Supplementary Table 4 Crude and adjusted ORs for adverse neonatal outcomes in singleton births by maternal albumin levels in women sampled at 12 weeks.**

|  | Q1 | P value | Q2 | Q3 | P value | Q4 | P value |
| --- | --- | --- | --- | --- | --- | --- | --- |
| PTB |  |  |  |  |  |  |  |
| OR (95% CI) | 0.86(0.72,1.03) | 0.109 | Reference | 1.18(0.99,1.41) | 0.061 | 1.02(0.84,1.25) | 0.819 |
| AOR (95% CI) | 0.82(0.68,0.99) | 0.034 | Reference | 1.23(1.03,1.47) | 0.021 | 1.08(0.88,1.32) | 0.476 |
| LBW |  |  |  |  |  |  |  |
| OR (95% CI) | 0.71(0.56,0.90) | 0.005 | Reference | 1.07(0.85,1.34) | 0.571 | 1.32(1.05,1.67) | 0.020 |
| AOR (95% CI) | 0.73(0.57,0.93) | 0.011 | Reference | 1.09(0.86,1.37) | 0.480 | 1.29(1.01,1.64) | 0.041 |
| Macrosomia | |  |  |  |  |  |  |
| OR (95% CI) | 1.13(0.97,1.32) | 0.117 | Reference | 0.94(0.79,1.11) | 0.454 | 0.74(0.61,0.91) | 0.003 |
| AOR (95% CI) | 1.02(0.87,1.20) | 0.779 | Reference | 0.97(0.82,1.15) | 0.704 | 0.81(0.66,0.99) | 0.035 |
| SGA |  |  |  |  |  |  |  |
| OR (95% CI) | 0.81(0.68,0.96) | 0.018 | Reference | 1.06(0.89,1.25) | 0.543 | 1.37(1.15,1.64) | <0.001 |
| AOR (95% CI) | 0.91(0.77,1.09) | 0.315 | Reference | 1.01(0.84,1.19) | 0.972 | 1.21(1.01,1.44) | 0.039 |
| LGA |  |  |  |  |  |  |  |
| OR (95% CI) | 1.28(1.15,1.42) | <0.001 | Reference | 0.91(0.81,1.02) | 0.111 | 0.75(0.66,0.85) | <0.001 |
| AOR (95% CI) | 1.16(1.04,1.29) | 0.008 | Reference | 0.94(0.84,1.06) | 0.335 | 0.82(0.72,0.94) | 0.005 |

Analyses were adjusted for age, BMI, gravidity, parity, educational level, alcohol and cigarette consumption before pregnancy, ALT, AST, pregnancy induced hypertension and gestational diabetes mellitus. OR Odd ratio, AOR adjusted odd ratio.
